# Reamer irrigator aspirator (RIA) reduces risk of fat embolism in bilateral pediatric femur shaft fractures: a case report

**DOI:** 10.1093/jscr/rjae042

**Published:** 2024-02-09

**Authors:** Erik Jacobson, Bailey C Schieve, Kyle J Klahs, Reuben A Macias, Amr Abdelgawad, Ahmed M Thabet

**Affiliations:** Pacific Northwest University-Health Sciences Medical School, Yakima, WA 98901, United States; Uniformed Services University Medical School, Bethesda, MD 20814, United States; Department of Orthopaedic Surgery, Texas Tech University Health Science Center, El Paso TX 79905, United States; Department of Orthopaedic Surgery, Texas Tech University Health Science Center, El Paso TX 79905, United States; Department of Orthopaedic Surgery, Maimonides Medical Center, Brooklyn, NY 11219, United States; Department of Orthopaedic Surgery, Texas Tech University Health Science Center, El Paso TX 79905, United States

**Keywords:** pediatric, femur fracture, bilateral femur fractures, Reamer Irrigator Aspirator (RIA), fat embolism syndrome

## Abstract

A 14-year-old male patient was successfully treated with the reamer irrigator aspirator for femur intramedullary rod preparation after sustaining right and left closed femur fractures because of an all-terrain vehicle accident. In patients already categorized as high risk for fat embolism syndrome, such as those with bilateral femur fractures, reaming both femora greatly increases the likelihood of this complication. The reamer irrigator aspirator provides an effective tool that potentially mitigates the risk of fat embolism syndrome in pediatric patients with this type of orthopedic trauma.

## Introduction

Bilateral femur fractures have a mortality rate of 5%–25%, and are an uncommon injury pattern in the pediatric population [1, 2]. Fat embolism syndrome (FES), which can develop secondary to long bone fracture, carries over a 30% mortality rate [3]. In patients with severe injury patterns such as bilateral femur fractures, an already high risk of mortality can be further compounded by an increased risk of developing FES.

The pathogenesis of FES is from intramedullary contents being extravasated into the systemic circulation. Clinical findings include hypoxia, tachypnea, altered mental status, petechial rash, acute respiratory distress syndrome, and even death [4, 5]. The etiology is theorized to be multifactorial but involves venous uptake and transportation of fat into the pulmonary capillary beds and brain where free fatty acids and chylomicrons trigger acute phase reactants [6–8]. Risk factors include closed and multiple fractures, young age, and prolonged conservative management of unstable long bone fractures [9].

To obtain appropriate intramedullary fit and to decrease delayed union, reaming is performed in long bone fractures prior to intramedullary nail (IMN) insertion [10, 11]. Reaming increases the amount of extravasated contents and theoretically FES [12]. In 2000, the reamer/irrigator/aspirator (RIA) (Synthes, Inc., West Chester, PA, USA) was introduced. It was designed as an intramedullary bone graft harvester for nonunion repair and arthrodesis. It was postulated that this device, which simultaneously reams, irrigates, and aspirates, could decrease the amount of debris from reaming, and was verified with an animal model study [13]. RIA is a single use item, listed at around $738 per set [14].

In this case report, we aimed to describe the risk stratification, and course of a 14-year-old male patient with bilateral femur fractures who was successfully treated utilizing the RIA. Such a case warrants particular attention to risk mitigation strategies as bilateral femoral fractures carry significantly higher risk of complications [1].

## Case report

**Figure 1:**
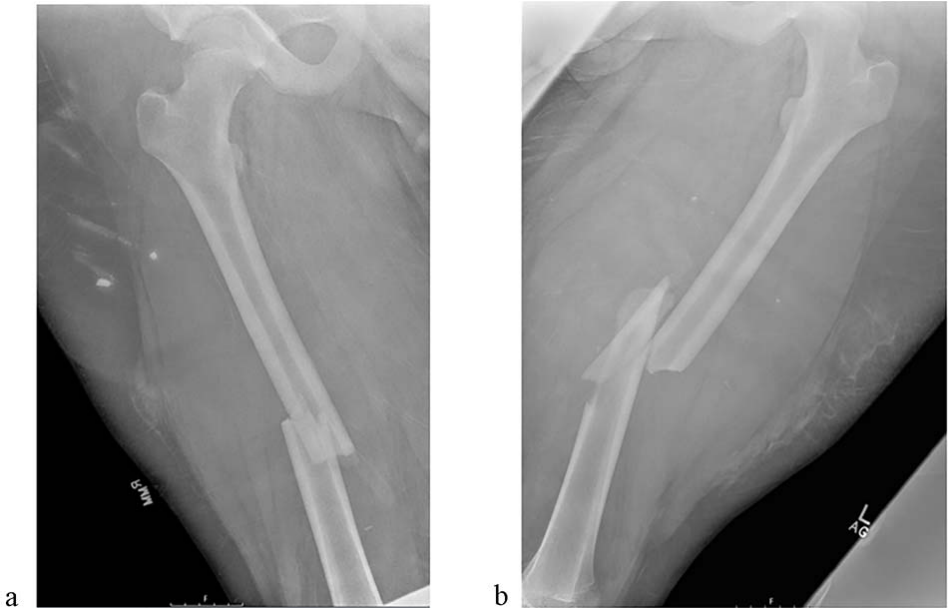
(a) Right femur fracture (OTA 32-A3). (b) Left femur fracture (OTA 32-B2).

**Figure 2:**
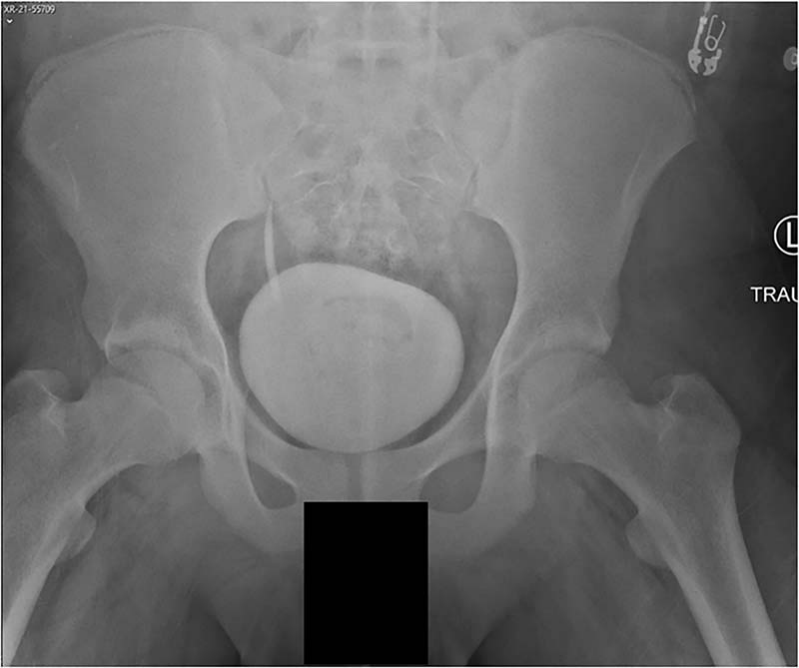
AP pelvis radiograph, modified Oxford Grade 25.

A 14-year-old boy sustained closed right and left mid-shaft femur fractures (Right OTA 32-A3, Left OTA 32-B2) after an all-terrain vehicle accident (Fig. 1a and b). The patient was classified as obese with a BMI of 37. Bone age was determined to be approaching skeletal maturity and so a rigid trochanteric entry femoral rod was selected for the implant [15, 16] (Fig. 2). Laboratory derangement included hyperphosphatemia and hypoalbuminemia. Therefore, additional measures to include early fracture stabilization and the RIA adjunct was selected for our patient [17].

**Figure 3:**
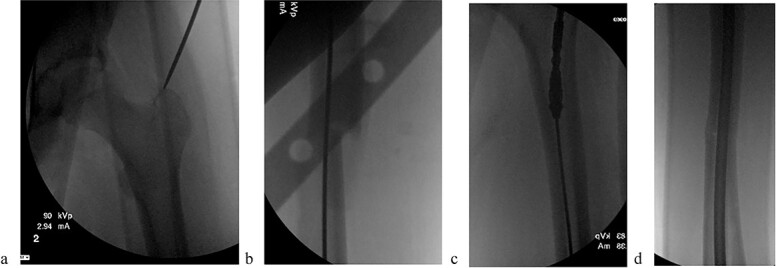
(a) Obtaining greater trochanteric entry starting point. (b) Fracture reduction with the “F” tool and passed ball-tipped guidewire. (c) Sequentially reaming with the RIA. (d) Maintained fracture reduction with implanted rigid femur rod.

The patient underwent sequential bilateral trochanteric entry antegrade femoral intramedullary rods (Stryker, Kalamazoo, MI, USA) after utilizing bilateral RIAs. We first passed the 8.5-mm end-cutting reamer from the RIA system (DePuy Synthes, Solothurn, Switzerland) and then upsized to an 11.5-mm reamer (Fig. 3a–d). We used the same reamers to prepare the contralateral side.

**Figure 4:**
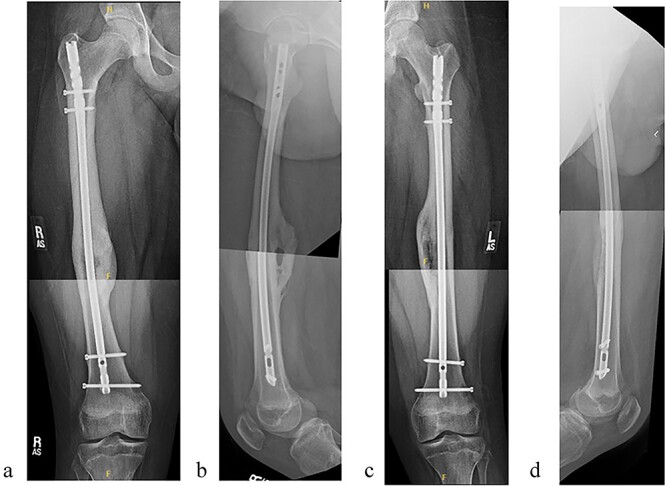
(a) Right femur AP radiograph. (b) Right femur lateral radiograph. (c) Left femur AP radiograph. (d) Left femur lateral radiograph.

**Figure 5:**
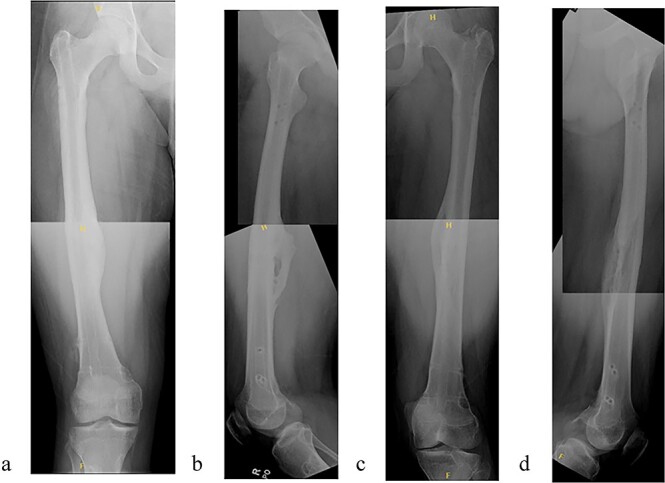
(a) Right femur AP radiograph after implant removal. (b) Right femur lateral radiograph after implant removal. (c) Left femur AP radiograph after implant removal. (d) Left femur lateral radiograph after implant removal.

The patient tolerated the procedure well and recovered uneventfully. He was noted to have circumferential osseous healing at 1 year and underwent bilateral femur IMN removal 1.5 years postoperative without complication (Figs 4a–d and 5a–d).

## Discussion

Advancements in reaming instrumentation design include fluted reamers, thinner diameter reamer shafts high-speed cutting reamers, and intramedullary lavage tubing; however, none have been shown to greatly decrease the risk of developing FES [6, 18]. The RIA has several novel design characteristics aimed at that task. First, the reamer head is single use, therefore does not blunt between cases. Irrigation of the canal during reaming decreases the overall temperature and lowers content viscosity. Aspiration allows for removal of reamed debris [19]. Hall *et al*. demonstrated that the RIA system reduced embolic events moderately as compared with their standard reaming group. In addition, RIA had a lower total emboli score compared with the standard reaming group [20]. However, they were unable to correlate this decrease in embolic score with any physiological changes. An animal study also showed that the number and the size of the microemboli in the unreamed (UR) and sequentially reamed (SR) groups were similar. But the RIA group had significantly fewer larger-sized (>200 μm) emboli compared with the UR group and the SR group [13].

It is important that orthopedic trauma patients be evaluated for the risk of developing FES. Lowery *et al*. determined that hypomagnesemia, hyperphosphatemia, hypoalbuminemia, blunt trauma mechanism of injury, and a greater number of bones fractured were risk factors [18]. Obesity and young age have also been established as risk factors for FES [21]. Based on these risk factors, our patient had an increased risk of developing FES and they were determined to be a suitable candidate for utilization of RIA.

While RIA may have potential use in treating shaft fractures in pediatric patients, there are some potential drawbacks with the system that have been studied. Traditional reaming is sequential to avoid too much reamer-canal size mismatch per pass. Jumping from initial to final diameter could increase the intramedullary pressure and facilitate the reamers getting stuck. It also allows the surgeon to judge the size of the medullary canal intraoperatively by determining when the reamer achieves endosteal contact. RIA requires either an established single-end size diameter preoperatively or open up multiple individual kits to achieve sequential reaming, which significantly contributes to expense. A meta-analysis by Laubach *et al*. found an overall low prevalence of complications (1.7%) with the RIA system. However, they note a steep learning curve in the use of the system. In addition, the main complications seen were cortex perforations and intraoperative blood loss which carried a blood transfusion rate of 9.72% [22].

This case report presented a 14-year-old with bilateral femur shaft fractures treated with reamed medullary nailing. The use of RIA to ream the medullary canal was effective in allowing nail placement and avoiding FES and ARDS. This is the first case report to utilize the RIA on a pediatric polytrauma patient. Further research is needed to further evaluate the safety and efficacy of the RIA in pediatric patients with complex orthopedic trauma.
